# microRNA-98 mediated microvascular hyperpermeability during burn shock phase via inhibiting FIH-1

**DOI:** 10.1186/s40001-015-0141-5

**Published:** 2015-04-23

**Authors:** Delin Hu, Youxin Yu, Chunhua Wang, Denghui Li, Yuncheng Tai, Linsen Fang

**Affiliations:** Department of Burns, The First Affiliated Hospital of Anhui Medical University, No. 218 Jixi Road, Hefei, Anhui Province 230022 People’s Republic of China

**Keywords:** Burn, FIH-1, miR-98, HIF-1α, Microvascular permeability

## Abstract

**Background:**

microRNA is a small non-coding RNA molecule and functions in RNA silencing and post-transcriptional regulation of gene expression. This study was designed to evaluate the role of miR-98 in the development of microvascular permeability and its molecular pathogenesis.

**Methods:**

Forty-eight healthy adult Wistar rats were divided into the control group (*n* = 8) and burn group (*n* = 40) that inflicted with 30% total body surface area third-degree burn. Groups were processed at 2, 4, 8, 12, and 24 h post-burn. Plasma for vascular endothelial cell culture was collected from control and 12 h post-burn rats. Organic microvascular permeability and serum miR-98 level were measured. *In vitro*, rat aorta endothelial cells were stimulated with burn serum. Level of miR-98 and protein of hypoxia-inducible factor-1 (HIF-1), factor inhibiting HIF-1α (FIH-1), and tight junction-associated proteins were determined.

**Results:**

Organic microvascular permeability began to rise at 2 h post-burn and maintained the same character throughout the experiment except in lung tissue that was still rising at 12 h; the serum level of miR-98 was elevated (*P* < 0.05). *In vitro*, burn serum stimulation increased rat aorta endothelial monolayer cell permeability as well as upregulated miR-98 expression (*P* < 0.05). As shown in the result of transfection experiment, miR-98 negatively regulated FIH-1 and tight junction-associated protein expression (*P* < 0.05).

**Conclusions:**

The findings of the present study suggest severe microvascular permeability due to burns; and the underlying mechanism bases on the promotion of miR-98 level to the extent that it activated HIF-1 gene expression, resulting in junction-associated protein deficiency.

## Background

The skin serves as a vital protective barrier against exterior harmful factors, and the disruption of its integrity can lead to patients’ significant disability or even death. Clinically, nearly 20 million annual hospital admissions are attributable to burns. Among these, the third-degree burn is the most threatening for injured patients and is characteristic of damage in all layers of skin, as well as muscles and possibly organs. Importantly, it is generally accepted that the burn injury-induced ischemic internal organs may contribute to the development of sepsis and multiple organ dysfunction [1-3], mainly including increased microvascular permeability [4]. Given this, better insight into the molecular mechanisms underlying pathogenic conditions related to microvascular hyperpermeability is required for developing effective therapeutic strategies for burn injury.

The integrity of microvascular permeability is mainly maintained by tight endothelial cell-cell junctions, which are composed of a large complex of proteins including the integral proteins such as claudins, occludin, and the peripheral membrane proteins such as zonula occludens (ZO-1 and ZO-2) [5,6]. Among these, ZO-1 is one of the most often investigated proteins and it is mainly responsible for connecting the integral membrane protein to the actin cytoskeleton and different types of signaling proteins [6]. Increasing evidences suggest that hypoxia-inducible factor-1 (HIF-1) that mediated adaptive cellular responses to hypoxia upregulates the expression and plays critical roles in paracellular barrier functions, including epithelial barrier [7-10]. HIF-1 protein consists of subunits HIF-1α and HIF-1β among which HIF-1α protein level is the dominant factor for HIF-1 transcriptional activity [11]. HIF-1-targeting genes are correlated with loss of ZO-1 and increased paracellular permeability [12-15]. Thus, efforts to attenuate the accumulation of HIF-1α may benefit burn patients who are at high risk for vascular organic permeability. In cells, the level and activity of HIF-1 are controlled by asparaginyl factor-inhibiting HIF-1 (FIH-1) hydroxylases. FIH-1 was originally found to be a negative regulator of HIF-1 and was later shown to be an asparaginyl hydroxylase, capable of hydroxylating N803 in the C-terminal activation domain (CAD) of human HIF-1 [16-18].

Although proteins have been established as basic regulatory factors in accommodation to alterations in environment, our knowledge of the contribution of regulatory micro-RNA molecules is gradually aggregating. As is known that microRNA is a small non-coding RNA molecule and functions in RNA silencing and post-transcriptional regulation of gene expression. Its important role in mediating the pathological process of ischemia has been identified. Especially, miR-98, a subset of HIF-1-inducible miRNAs, was found expressed to the greatest extent during ischemia [19]. In this study, we attempt to investigate whether miR-98 is involved in burn injury-induced vascular permeability and to investigate the molecular mechanism underlying burn-induced microvascular hyperpermeability in both rat model of burn injury and microvascular endothelial monolayer model of burn serum triggering.

## Methods

### Animal models

The protocol was approved by the Committee on the Ethics of Animal Experiments of the Anhui Medical University of Science and Technology. Male Wistar rats were obtained from the Institute of Laboratory Animal Resources at Anhui Medical University (8 to 10 weeks, weighing 271 ± 10 g) and were housed in mesh cages in a room maintained at 25°C, illuminated with 12:12-h light-dark cycles and provided with standard rodent chow and water *ad libitum*. For experiment, 48 mice were randomly divided into two groups: control group (*n* = 8) and burn operation group (*n* = 40). Full-thickness burn injury occupying 30% of total body surface area was produced as described by Ikezu *et al* [20]. After the rat was deeply anesthetized with pentobarbital sodium (25 mg/kg), the hair on its back was removed with electric hair clippers and the skin was depilated with 8% sodium sulfide. Rats were subjected to scald injury by smearing with 3% napalm for burning 18 s. The operation resulted in 30% total body surface area (TBSA) III degree burns. Preliminary histological investigation established the presence of a deep partial-thickness burn wound using the described protocol (data not shown). Burn-mice were intraperitoneally injected with Lactated Ringer’s Solution (40 ml/kg) for anti-shock. Following burn injury, animals received a subcutaneous injection of 0.5 ml normal saline with 0.1 mg/kg of buprenorphine (Sigma, St. Louis, MO, USA) for pain control. Mice in the control group were treated in proportion except burn injury operation. Burned rats did not display discomfort or pain, and all rats consumed food and water within 45 min of the burn procedure.

Plasma was harvested from either control rats or burned-rats 2, 4, 8, 12, and 24 h post-injury (8 rats per time phase). The rats were deeply anesthetized with methoxyflurane, and aorta abdominalis was used to collect blood into plasma separator tubes containing lithium heparin (Microtainer, Becton, Dickinson and Company, Franklin Lakes, NJ, USA). The tubes were then centrifuged at 200 *g* for 10 min. The plasma was aliquoted and stored at −80°C for miR-98 detection. Additionally, plasma from control and 12-h post-burn rats was collected and was performed with 56°C water-bath for 30 min in order to inactivate serum complements and then filtrated with a 0.22-um filter membrane. The plasma was finally aliquoted and stored at −80°C for vascular endothelial cell culture.

### Organ microvascular permeability assay

For the permeability assay, vascular protein leakage was measured using the Evans blue (EB) technique. After deep anesthetization, EB (20 mg/kg weight; Sigma, St. Louis, MO, USA) was injected intravenously through the femoral vein. Thirty minutes after dye injection, the animals were euthanatized, and a midline thoracotomy was performed. Then the superior and inferior vena cavae were ligated, the aorta was transected, and 20 ml of NS was injected into the right ventricle at a pressure of 20 cm H_2_O to wash out the internal organs’ intravascular content. A sample of lung, liver, ileum, and kidney tissue was weighed, immersed in N,N-dimethylformamide (Sigma), and homogenized. The homogenate was incubated at room temperature for 48 h. Eluted EB was measured at 620 nm using an automatic microplate reader (SpectraMax M5; Molecular Devices, Sunnyvale, CA, USA), and the amount was expressed as micrograms per 100 mg dry tissue.

### Endothelial cell culture and treatment

Rat aortic endothelial cells (RAECs) were purchased from Genlantis (San Diego, CA, USA). RAECs maintained at 37°C in 5% CO_2_ in Dulbecco’s Modified Eagle’s Medium (DMEM; Hyclone Laboratories, Logan, UT, USA) containing 10% fetal bovine serum. Cells at passages 3 to 10 were used in this study. For subsequent study, confluent cultures were stimulated with burn serum (10% of DMEM) for 24 h accompanied by normal serum incubation as control.

### Transfections with miRNA mimics and inhibitors

Rat aortic endothelial cells (2 × 10^5^) were transfected with 10 to 50 nM anti-miR-98 miRNA inhibitors (Ambion, Austin, TX, USA) or scrambled control (Negative Control 1; Ambion) by using Lipofectamine 2000 from Invitrogen (Carlsbad, CA, USA) according to the manufacturer’s protocol.

### Quantitative real-time PCR (qRT-PCR)

For miR-98 expression analysis, total RNAs were extracted with Trizol reagent (Invitrogen, Carlsbad, CA, USA) according to the protocols of the manufacturer followed by DNA-free DNase treatment (Ambion, Austin, TX, USA). The total RNAs were inversely transcribed into cDNA using the Revert Aid™ First Strand cDNA Synthesis Kit (Thermo Scientific, Grand Island, NY, USA) according to the manufacturer protocols. For amplification, the PCR was performed in a total reaction volume of 20 ml with 5 μl of cDNA template, 500 nM forward primer, 500 nM reverse primer, and 10 μl of SsoFast™ EvaGreen® Supermix (Bio-Rad, Mississauga, ON, USA) and was completed in the ABI PRISM 7000 Fluorescent Quantitative PCR System (Applied Biosystems, Foster City, CA, USA). U6 was used as an internal control.

### Transendothelial cell electric resistance

All resistance measurements were conducted by an epithelial voltohmmeter (World Precision Instruments, Sarasota, FL, USA) with STX2 chopstick electrodes (World Precision Instruments). A 12-well tissue culture plate (Sigma, St. Louis, MO, USA) was inserted with 12-mm transwell inserts that have been coated with a solution of human fibronectin (Sigma, St. Louis MO, USA) dissolved in PBS at a concentration of 50 μg/ml for 6 h at 25°C. After pre-treated with 10 μl burn serum or control serum in DMEM complete medium for 24 h, RAECs with the incubating medium were plated in the multiwall inserts and then cultured for an additional 5 to 7 days until the transendothelial electric resistance (TEER) was ≥20 ohm × cm^2^. In other experiments, using the same culture conditions to establish TEER ≥ 20 ohm × cm^2^, RAECs were transfected with miR-98 inhibitor and/or control siRNA or sequence specific siRNA directed against FIH-1 in 10% fetal bovine serum (FBS) for 24 h.

### Western blot assay

The lung tissues were homogenized and analyzed for HIF-1α, FIH-1, and junction-associated proteins by Western blotting. Protein concentrations were determined using the bicinchoninic acid method. An equal amount of protein was loaded onto 10% sodium polyacrylamide hydrophilic gel for electrophoretic separation. After electrophoresis, proteins were electroblotted onto polyvinylidene fluoride (PVDF) membranes (Millipore, Billerica, MA, USA) and blocked in 10% nonfat milk. Following, the proteins were blotted with primary antibodies against HIF-1α, FIH-1, Occludin, Claudin-1, and ZO-1 (Abcam, Cambridge, United Kingdom) and β-actin (Tianjin Sungene Biotech Co., Ltd., Tianjin, China) at 4°C overnight. The next day, the blots were recognized by horseradish peroxidase-tagged secondary antibody (Santa Cruz, CA, USA) incubation. Finally, the protein expressions were represented using an enhanced chemiluminescence method, and the images were visualized using ChampGel (Sage Creation Science Co, Beijing, China).

### Permeability coefficient of albumin

The permeability of the endothelial cell monolayer was performed with a two-compartment system that was separated by a filter membrane. Briefly, both compartments contained that same basal medium modified Tyrode’s solution (composition in mM: 150 NaCl, 2.7 KCl, 1.2 KH2PO4, 1.2 MgSO4, 1.0 CaCl2, and 30.0 N-2-hydroxyethylpiperazine-N′-2-ethanesulfonic acid; pH 7.4, 37°C) supplemented with 2% (vol/vol) NCS and were with no hydrostatic pressure gradient. The monolayer in a ‘luminal’ compartment possessed a volume of 2.5 ml and that in an ‘abluminal’ compartment possessed a volume of 6.5 ml. The medium in the abluminal compartment was constantly stirred. The luminal compartment was added with trypan blue-labeled albumin (60 μM). The appearance of the labeled albumin in the abluminal compartment was continuously monitored by pumping the liquid through a spectrophotometer (Specord 10; Carl Zeiss, Thornwood, NY, USA). The concentration of labeled albumin in the luminal compartment was determined every 10 min of incubation. It did not change significantly in the time frame of the experiments.

The albumin across the monolayer with the surface were represented as permeability coefficient (Pa): Pa = ([*A*]/*t*) × (*l*/*A*) × (*v*/[*L*]). [*A*] is the luminal compartment albumin concentration, *t* is the time interval showed in seconds, *A* is the area of glomerular capillary membrane showed in cm^2^, *v* is the fluid volume of abluminal compartment showed in ml, [*L*] is albumin concentration of abluminal compartment. The result was presented as percentage change of Pa: Equation Pa% = (test Pa/control Pa) × 100.

### miRNA target reporter luciferase assay

MiR-98 predication of potential targets FIH-1 3'UTR was performed with miRanda software. Fragments of 3'UTR of FIH-1 gene harboring the predicted miR-98-binding sites were cloned into the firefly luciferase reporter plasmid pMIR-Report (Ambion) according to the manufacturer’s protocol. After 24 to 48 h transfection, cells were harvested and lysed, and luciferase reporter activities were measured using Dual-Glo® Luciferase Assay System (Promega, Madison, WI, USA). Firefly luciferase activity was normalized to Renilla luciferase activity and total protein, determined using the bicinchoninic acid (BCA) protein assay kit. Values for cells without miRNA mimic transfection were set equal to 1.

### Luciferase activity

Rat aortic endothelial cells (2 × 105) were stably transfected with HIF-1α luciferase reporter (RC0017, Panomics, Fremont, CA, USA) and seeded in complete media (DMEM with 10% FBS) in 24-well plates. Luciferase activity was measured on hour 24 with HIF-1 alpha Transcription Factor Assay (ab133104; Abcam, Cambridge, MA, USA) according to protocol. In brief, cells were lysed by each well being added with cell lysis solution. Then, lysates mixed with ATP mix served as the substrate for the chemiluminescent oxidation-reduction reaction which leads to light emission and oxy-luciferin generation. The reaction liquid was measured with a luminometer at a light of 560 nm.

### Statistical analysis

All of the experiments were performed with samples in triplicate or greater, and the data are presented as the mean ± standard deviation (SD) of observations. The experimental groups were compared by one-way or two-way analysis of variance followed by a *post hoc* Tukey’s test. Differences were considered statistically significant if the *P* value was less than 0.05.

## Results

### Changes in organic microvascular permeability in burn-injured rat

**Figure 1:**
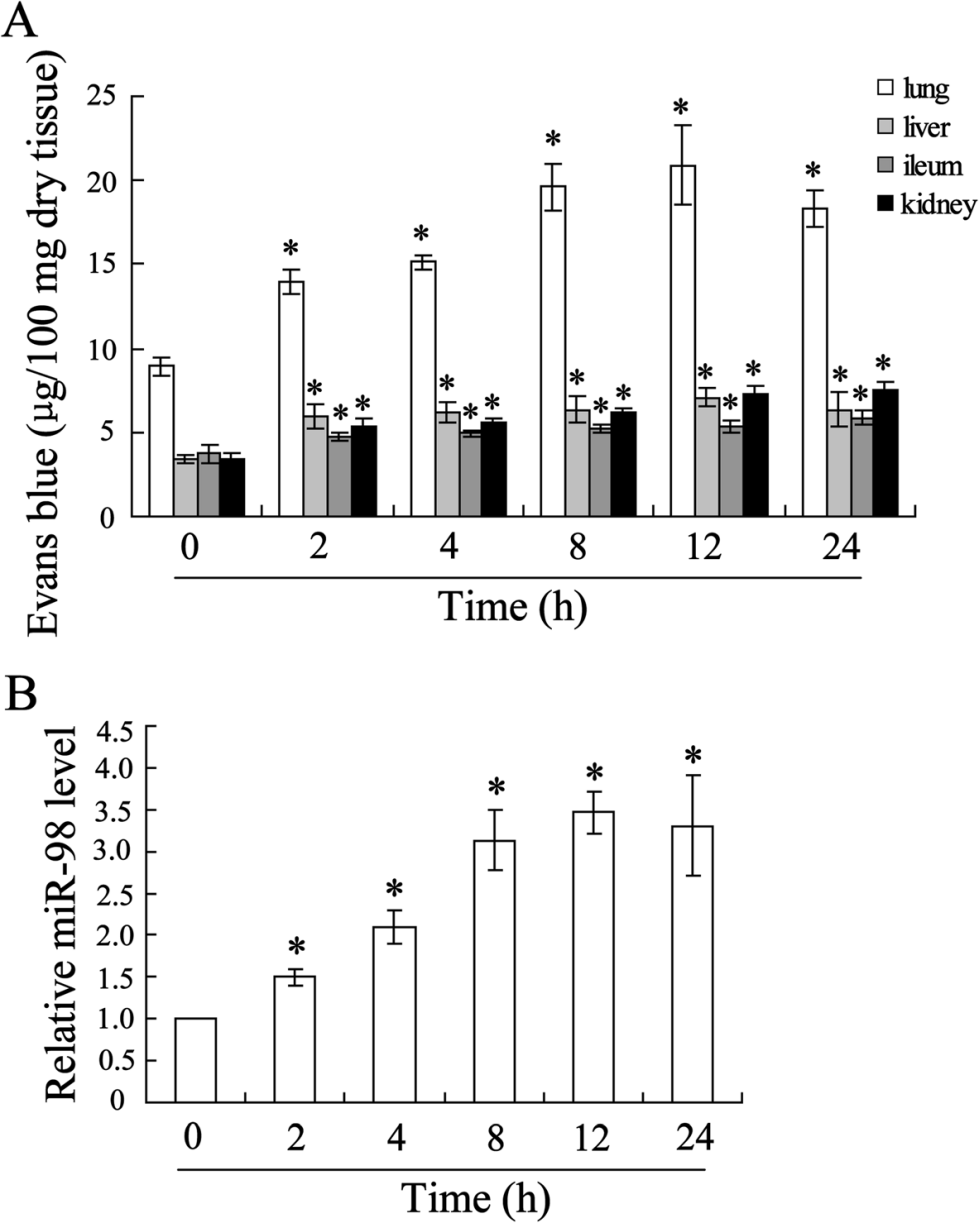
Burns induced increase of vascular permeability. Mice burn model was established, and then on different time points after surgery, mice were euthanatized for the following detection. **(A)** Vascular permeability was evaluated using Evans blue contents in tissues. **(B)** Levels of miR-98 in serum was determined using quantitative real-time PCR. The data were represented with mean ± SD. ^*^ *P* < 0.05 compared with corresponding control.

We assessed changes in organic vascular permeability in mice model of burn using Evans blue assay. The Evans blue contents were determined regularly and began to increase significantly as early as hour 2, peaked at hour 12, and sustained a high level in lung tissues. While in tissues of the liver, ileum, and kidney, compared with no burn, Evans blue contents were also increased at hour 2, but maintained the same level in the following 22 h (Figure 1A). Level of serum miR-98 was evaluated, and the data showed an increase in a time-dependent manner and peaked on hour 12 (Figure 1B).

### Burn serum stimulation increased permeability of rat aorta endothelial cell monolayers

**Figure 2:**
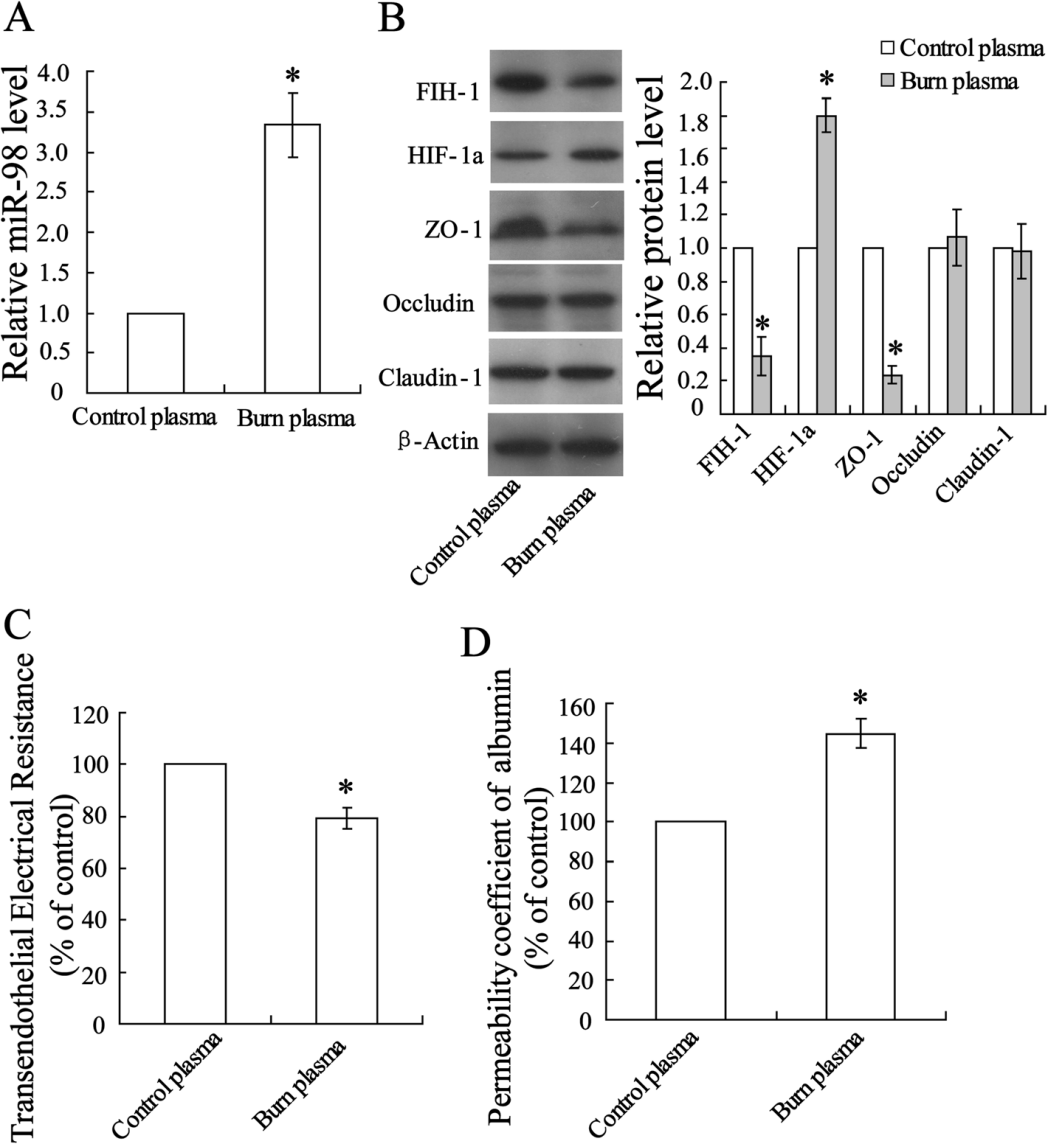
Burn serum stimulation increased permeability of rat aorta endothelial cell monolayers. Rat aorta endothelial cells were incubated with burn serum for 12 h. **(A)** Level of miR-98 expression was determined using quantitative real-time PCR. **(B)** Profiles of tight junction-associated proteins expression were evaluated using Western blot. **(C)** Transendothelial electrical resistance and **(D)** permeability coefficient of albumin were performed with monolayer endothelial cells. The data were represented with mean ± SD. ^*^ *P* < 0.05 compared with control plasma.

To investigate the possible mechanism underlying burn-induced microvascular permeability, *in vitro* burn injury models of rat aorta endothelial monolayer cells stimulated with burn serum were utilized in the experiment. Incubation cells with burn serum led to miR-98 upregulation (Figure 2A) that corresponds to the observation in Figure 1B. It has been well accepted that an insufficient amount of tissue perfusion after burn results in ischemia anoxic damage of cells [21]. Thus we detected protein expression of hypoxia-inducible factor-1 (HIF-1) and factor inhibiting HIF-1α (FIH-1). As shown in Figure 2B, burn serum stimulation increased FIH-1 expression while decreased HIF-1α expression. Following, profiles of tight junction-associated proteins were evaluated and the results showed there is no change in Occludin and Claudin-1 expression except ZO-1. The permeability of endothelial cell monolayers was evaluated using transendothelial electrical resistance assay (Figure 2C) and permeability coefficient of albumin (Figure 2D). Burn serum reduced transendothelial electrical resistance and increased albumin permeability indicating increased permeability of the endothelial cell.

### miR-98 negatively regulated FIH-1 expression in rat aorta endothelial cell

**Figure 3:**
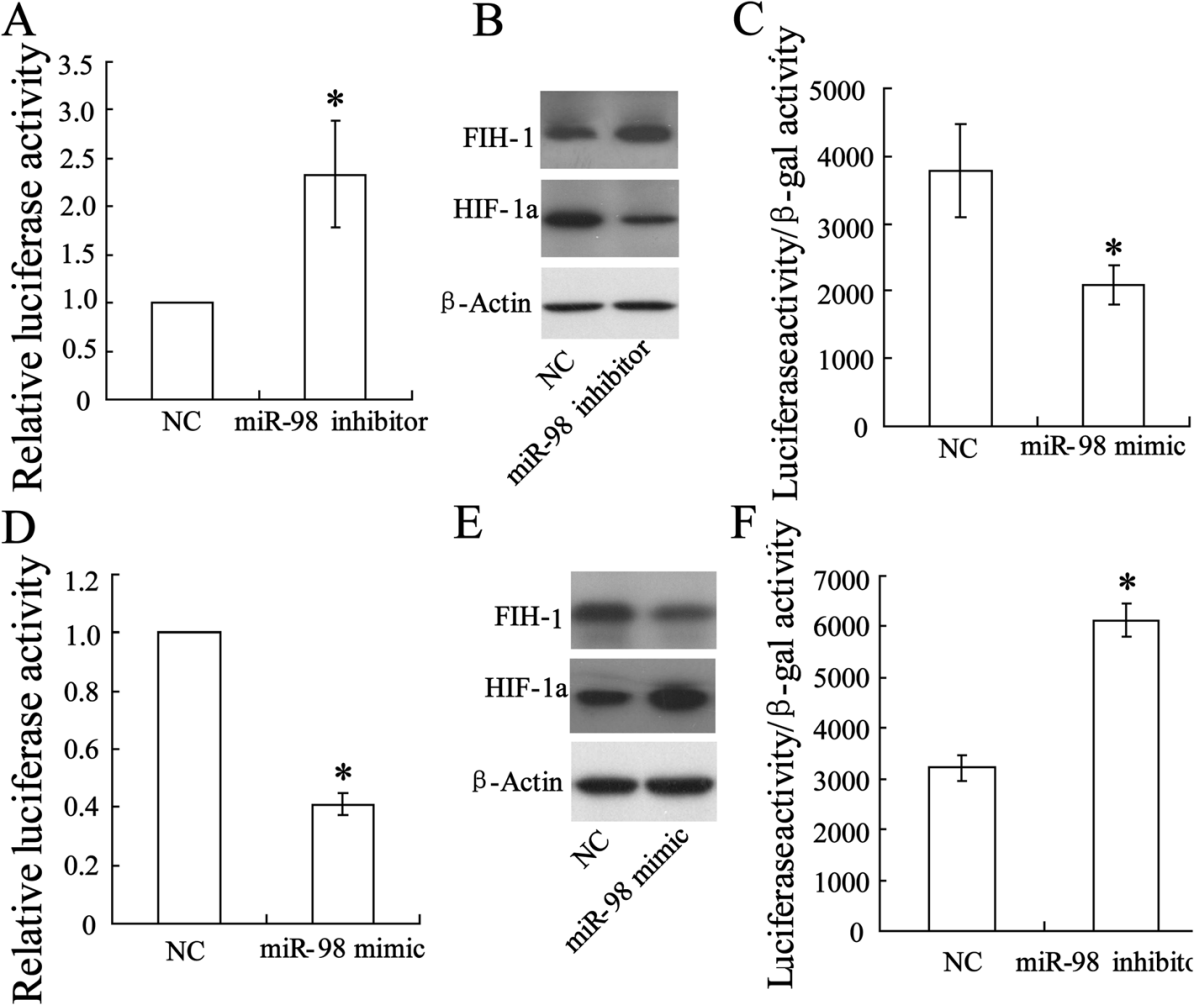
Regulated role of miR-98 in FIH-1 expression in rat aorta endothelial cell. Knock down of miR-98 by miR-inhibitor **(A)** increased FIH 3ʹUTR activity, **(B)** suppressed HIF-1 expression, and **(C)** inactivated HIF-1 transcription. Overexpression of miR-98 by miR-98 mimic transfection **(D)** inhibited FIH 3ʹUTR activity, **(E)** promoted HIF-1 expression, and **(F)** increased HIF-1 transcription activity. The data were represented with mean ± SD. ^*^ *P* < 0.05 compared with negative control.

To investigate the regulating role of miR-98 in FHI-1 expression in rat aorta endothelial cell, miR-98 was knocked down by cells transfected with miR-98 inhibitor. Forty-eight hours after transfection, FIH 3'UTR activity was elevated (Figure 3A) and FIH-1 protein level was upregulated, while HIF-1 protein was downregulated (Figure 3B). HIF-1α transcription factor activity was attenuated (Figure 3C). When miR-98 was overexpressed by miR-98 mimic transfection, FIH 3'UTR activity was decreased (Figure 3D) and FIH-1 protein level was downregulated, while HIF-1 protein was upregulated (Figure 3E). Consequently, HIF-1α transcription factor activity was enhanced (Figure 3F).

### Silenced miR-98 abrogated burn serum-increased permeability in rat aorta endothelial cells

**Figure 4:**
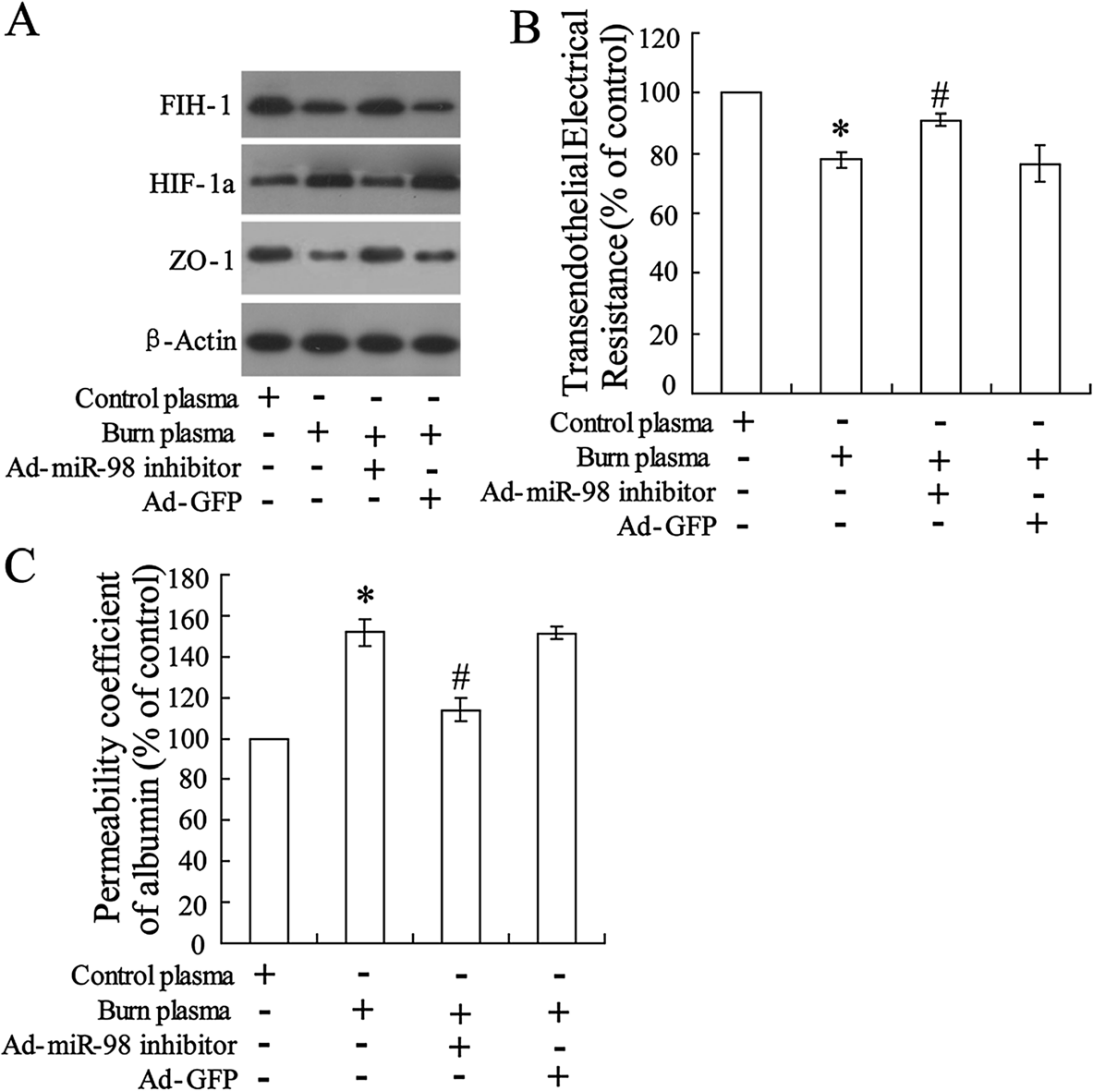
Silenced miR-98 abrogated burn serum-increased permeability in rat aorta endothelial cells. After transfected with miR-98 inhibitor for 48 h, cells were incubated with burn serum or normal serum (as control) followed. **(A)** Profiles of protein expression were determined using Western blot. **(B)** Transendothelial electrical resistance and **(C)** permeability coefficient of albumin were examined with monolayer endothelial cells. The data were represented with mean ± SD. ^*^ *P* < 0.05 compared with cell incubated with control plasma; ^#^ *P* < 0.05 compared with cell incubated with burn plasma.

Rat aorta endothelial cells were pretreated with miR-98 inhibitor and then stimulated with burn serum. Protein levels are represented by Western blot in Figure 4A and the result showed a reversing effect of silenced miR-98 on burn serum-reduced FIH-1 expression and burn serum-induced FIH-1 expression as well as burn serum-reduced ZO-1 expression. Additionally, the permeability of endothelial cell monolayers was evaluated using transendothelial electrical resistance assay (Figure 4B) and permeability coefficient of albumin (Figure 4C). The results also showed the abrogating effect of silenced miR-98 on burn serum-reduced transendothelial electrical resistance and increased albumin permeability suggesting a protective effect of silenced miR-98 on endothelial cell monolayer permeability.

### Knock down FIH-1 abrogated silenced miR-98-reduced permeability in rat aorta endothelial cells

**Figure 5:**
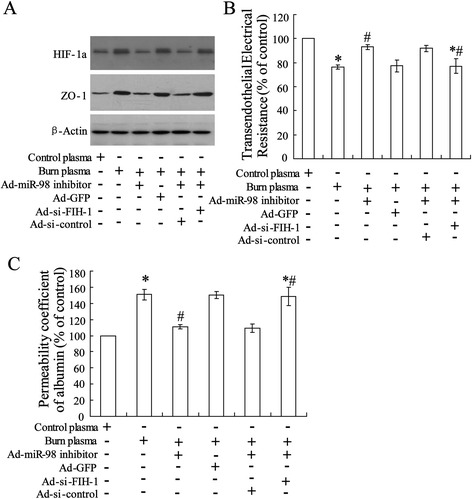
Co-silenced FIH-1 abrogated silenced miR-98-reduced permeability in rat aorta endothelial cells. Cells were pretreated with siRNA-FIH-1 followed by transfection with miR-98 inhibitor. After 48 h, the cells were incubated with burn serum or control serum. **(A)** Profiles of protein were determined using Western blot. **(B)** Transendothelial electrical resistance and **(C)** permeability coefficient of albumin were performed with monolayer endothelial cells. The data were represented as mean ± SD. ^*^ *P* < 0.05 compared with control plasma; ^#^ *P* < 0.05 compared with burn serum; ^*#^ *P* < 0.05 compared with burn serum + miR-98 inhibitor.

In view of our previous observations that miR-98 inhibitor treatment can increase FIH-1 expression and reversed burn-induced permeability, we next investigated the outcome of siRNA-mediated gene depletion of HIF-1 on the silenced miR-98-reduced monolayer endothelial cell permeability. As shown in Figure 5A, co-transfected miR-98 inhibitor with siRNA-FIH-1 reversed silenced miR-98-reduced ZO-1 expression. The permeability of endothelial cell monolayers was evaluated. The results showed that the abrogating effect of silenced miR-98 on burn serum-reduced transendothelial electrical resistance was inhibited by siRNA-FIH-1 treatment (Figure 5B). And abrogating effect of silenced miR-98 on burn serum-increased albumin permeability was also suppressed (Figure 5C).

## Discussion

**Figure 6:**
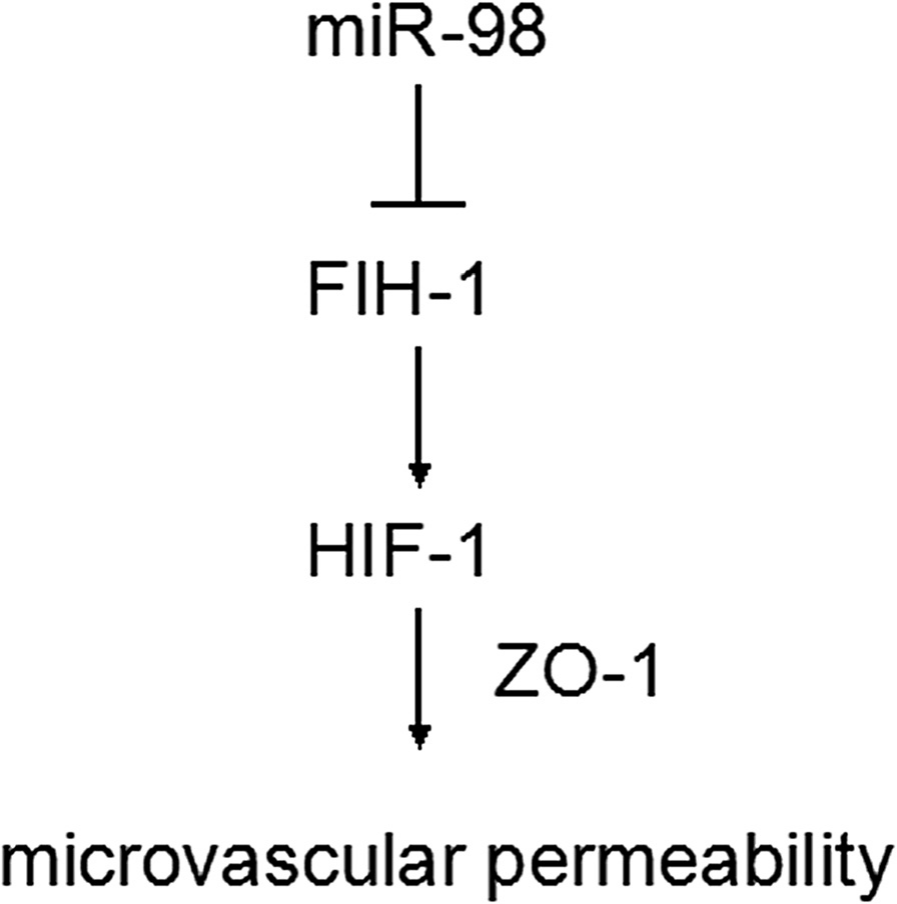
Signaling mechanism by which miR-98 mediated burn-induced increase of microvascular permeability.

Increased organic microvascular hyperpermeability induced by severe burns is a fatal threat for patient survival. In this study, we attempted to explored the innate underlying mechanism of burn-triggered microvascular hyperpermeability change during burn injury and explore potential therapy target (Figure 6). From the result, we found out that miR-98 are the key regulators in burn-induced internal organ endothelial dysfunction via inhibiting FIH-1 expression. Our data support that major burn injury induces miR-98 accumulation, which suppressed FIH-1 gene transcription, leading to loss of tight junction-associated ZO-1 protein and the subsequent increase in lung microvascular barrier permeability. Thus, our results provided the possible therapeutic target that elevated FIH-1 by inhibiting miR-98 results in accumulation of HIF-1-mediated adaptive cellular responses to burn-induced ischemia and upregulation of the expression of junction-associated genes that protects against burn-induced internal organ epithelial barrier dysfunction.

Burn injury is a common trauma, and the internal organs are some of the most vulnerable distant organs that are affected by extensive burns [22]. Recent studies have shown that pathophysiology of burn-induced internal organ damage is derived from endothelial cell dysfunction [23]. This symptom was also presented in our *in vivo* experiments in which burn stimuli significantly increase microvascular permeability of the liver, ileum, kidney, and lung. It is well known that, as a semipermeable barrier, endothelial cell in vascular actively participates in blood-tissue exchange of plasma fluid, proteins, and cells and is essential for maintaining circulatory homeostasis [24] that plays an important role in burn patients’ survival. An extensive research on a possible mechanism underlying endothelial cell permeability increase is urgently needed.

The decreased permeability is usually found in hypoxic-ischemic organs [25]. During ischemia, many inflammatory mediators are locally accumulated and capable of disrupting the interendothelial junction assembly, thereby causing endothelial hyperpermeability. Usually, adaptive cellular responses to hypoxia are mediated by HIF-1. Notably, the main burn-induced organ damage is clinically characterized by hypoxemia that activates HIF-1-mediated adaptive cellular responses [26]. Incubating endothelial cells with burn serum, we found an increased HIF-1α expression that was forcefully proved by decreased FIH-1 expression that negatively controlled cellular level and activity of HIF-1 under hypoxia [16]. Given this, both positive and negative regulators of FIH-1 provide important potential therapeutic targets for drugs that can be used to manipulate HIF-1 expression in burn pathological conditions.

Due to its ubiquity in mammalian, miRNAs are increasingly explored in various body functions. Importantly, alterations in their levels may compromise cellular function. Accumulating evidence focuses on regulating the role of miRNA on the function of protein expression under cell hypoxic-ischemic conditions [27,28]. Among these, miR-98 was found expressed to the greatest extent during hypoxia in squamous cell carcinoma [14]. Interestingly, we found upregulated miR-98 in rat models of burn and burn serum-stimulated rat vascular endothelial cells. We hypothesized the possible involvement of miR-98 in acute post-burn pathophysiologic process. Importantly, we identify the possible target FIH-1 by miR-98 by result of fluorescent report assay which was further confirmed by protein expression assay showing that silenced miR-98 promoted FIH-1 expression followed with a decreased HIF-1 protein level. These data strongly verify our hypothesis on the possible involvement of miR-98 in acute post-burn pathophysiologic process. In view of the above result, expression change of miR-98 would influence burn-induced vascular hyperpermeability. This was supported by monolayer endothelial cell analysis showing that silenced miR-98 reversed burn serum-induced junction-associated ZO-1 protein as well as hyperpermeability.

## Conclusions

In summary, severe burn injury-induced internal organic hyperpermeability was mediated by upregulated miR-98 expression that inhibited FIH-1 protein expression to the extent that it activated HIF-1 gene expression, resulting in junction-associated protein deficiency. So, targeting miR-98 that mediate FIH-1 gene expression suppression demonstrates a therapeutic potential to improve vascular barrier function during burn injury.

## Abbreviations

BCA: Bicinchoninic acid
CAD: C-terminal activation domain
DMEM: Dulbecco’s Modified Eagle’s Medium
EB: Evans blue
FIH-1: Factor-inhibiting HIF-1
HIF-1: Hypoxia-inducible factor-1
NIH: National Institutes of Health
PVDF: Polyvinylidene fluoride
RAECs: Rat aortic endothelial cells
SD: Standard deviation
TBSA: Total body surface area
TEER: Transendothelial electric resistance
-1 and ZO-2: Zonula occludens
